# Research on RNA secondary structure predicting via bidirectional recurrent neural network

**DOI:** 10.1186/s12859-021-04332-z

**Published:** 2021-09-08

**Authors:** Weizhong Lu, Yan Cao, Hongjie Wu, Yijie Ding, Zhengwei Song, Yu Zhang, Qiming Fu, Haiou Li

**Affiliations:** 1 School of Electronic and Information Engineering, Suzhou University of Science and Technology, Suzhou, 215009 China; 2 Jiangsu Province Key Laboratory of Intelligent Building Energy Efficiency, Suzhou University of Science and Technology, Suzhou, 215009 China; 3 Suzhou Industrial Park Institute of Services Outsourcing, Suzhou, 215123 China

**Keywords:** Recurrent neural network, RNA secondary structure prediction, Pseudoknots

## Abstract

**Background:**

RNA secondary structure prediction is an important research content in the field of biological information. Predicting RNA secondary structure with pseudoknots has been proved to be an NP-hard problem. Traditional machine learning methods can not effectively apply protein sequence information with different sequence lengths to the prediction process due to the constraint of the self model when predicting the RNA secondary structure. In addition, there is a large difference between the number of paired bases and the number of unpaired bases in the RNA sequences, which means the problem of positive and negative sample imbalance is easy to make the model fall into a local optimum. To solve the above problems, this paper proposes a variable-length dynamic bidirectional Gated Recurrent Unit(VLDB GRU) model. The model can accept sequences with different lengths through the introduction of flag vector. The model can also make full use of the base information before and after the predicted base and can avoid losing part of the information due to truncation. Introducing a weight vector to predict the RNA training set by dynamically adjusting each base loss function solves the problem of balanced sample imbalance.

**Results:**

The algorithm proposed in this paper is compared with the existing algorithms on five representative subsets of the data set RNA STRAND. The experimental results show that the accuracy and Matthews correlation coefficient of the method are improved by 4.7% and 11.4%, respectively.

**Conclusions:**

The flag vector introduced allows the model to effectively use the information before and after the protein sequence; the introduced weight vector solves the problem of unbalanced sample balance. Compared with other algorithms, the LVDB GRU algorithm proposed in this paper has the best detection results.

## Background

Ribonucleic acid(RNA), as the genetic carrier of living organisms, plays a very important role in living organisms, especially in HIV and other viruses, its genetic information is carried by RNA rather than DNA [1]. The function of RNA is usually determined by its spatial structure, which is usually divided into three levels. The primary structure of RNA refers to the arrangement order of four nucleotides. Because different bases cause different nucleotides, the primary structure of RNA is represented by four bases: A, C, G and U. The secondary structure of RNA refers to the planar structure formed by the interaction and folding of non-adjacent bases. Hairpin loop, bulge loop, inner loop, multi-branched loop, single-stranded regions, helix and pseudoknots are seven recognized secondary structural elements. At present, a large number of experiments have shown that the secondary structure of RNA is closely related to its function [2]. Therefore, studying the secondary structure of RNA is the first step for us to understand and study its function [3]. However, RNA molecules have the characteristics of difficult crystallization and fast degradation, the traditional methods of X-ray crystal diffraction and nuclear magnetic resonance to determine the secondary structure are not only time-consuming, costly and expensive, but also not suitable for all RNA molecules [4].

At present, the prediction of RNA secondary structure is mainly divided into three categories: methods based on minimum free energy, methods based on sequence comparison and methods based on statistics. The minimum free energy model is generally used to predict the secondary structure of RNA under the condition that only the primary sequence of RNA does not have any prior knowledge [5, 6]. This model assumes that RNA will fold into a stable secondary structure with minimum free energy. Based on this idea, Akiyama et al. proposed a weighted method combining thermodynamic method and machine learning [7]. This method avoids the over-fitting problem that may occur in the original model by adding regularization terms in the training process on the basis of the original machine learning model. Islam et al. proposed a model based on chemical reaction optimization algorithm (CRO) [8]. The model accelerates the prediction time to a certain extent by verifying and deleting repetitive stems into RNA sequences. Jin Li et al. proposed an RGRNA model based on stem replacement and growth, which improves the accuracy of RNA secondary structure prediction by using a combination optimization algorithm [9]. However, these methods still have two shortcomings: first, their prediction accuracy is relatively low, usually only between 50 and 70%; second, this method only considers the effect of pairing base pairs on free energy, it cannot predict RNA sequences with pseudoknots. However, pseudoknots are common structures in RNA sequences, so this method has obvious limitations. The method based on comparison sequence is to determine the secondary structure of RNA by comparing and analyzing a large number of homologous RNA molecular sequences. TurboFold II proposed by Zhen Tan et al. is a way to predict RNA secondary structure based on multiple RNA homologous sequences. Compared with TurboFold [10], TurboFold II increases the comparison of multiple sequences. The aliFreeFold model proposed by Ouangraoua et al. is a method to speed up the prediction by calculating a suboptimal secondary structure generated by a representative structure for each sequence from a group of homologous RNA sequences when the number and divergence of homologous RNA increase and the prediction effect is not ideal. Among these methods, the method of comparison before prediction is based on the premise that the structural conservativeness is greater than the sequence conservativeness. The prediction effect of this method strongly depends on the results of sequence comparison. The main idea of the simultaneous prediction and sequence comparison method is to cycle the sequence comparison and maximum base pair folding, which consumes the time and space resources of the computer. The method of prediction before comparison takes evolutionary information into account, but this method cannot predict RNA sequences with pseudoknots. Based on the idea of statistics, the problem can be transformed into the classification problem of base pairing results in sequences. Bellaousov S et al. proposed the ProbKnot method to predict the secondary structure of RNA by comparing the predicted structure and known structure of a large RNA sequence database containing less than 700 nucleotides [11]. Rujira Achawanantakun et al. used the method of preserving the adjacency and nesting of structural features without considering the abstract shape of spiral and loop region length details, and used the method of support vector machine to predict the secondary structure of RNA, which can predict RNA sequences containing pseudoknots [12]. These algorithms have also achieved certain results, but there are some deficiencies. First, RNA base pairing is a complex biological process, and it is difficult to mine the information contained in the sequence by a simple formula or a shallow level of regular learning [13, 14]. Second, they are limited by the problem of their own model so that they can only accept fixed-length sequence information [15]. Third, due to the imbalance of positive and negative samples in the training data set, the trained model is likely to be locally optimal (Table 1).

**Table 1:**
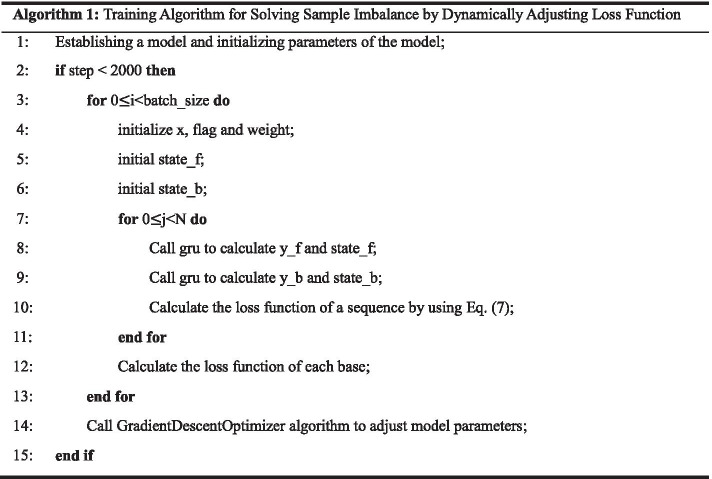
Training algorithm for dynamically adjusting loss function

In this paper, a variable-length dynamic bidirectional Gated Recurrent Unit(VLDB GRU) model is proposed to solve the above problems according to the characteristics of current RNA secondary structure prediction methods and their defects [16, 17]. The prediction of RNA secondary structure is based on sequence, namely, the force of hydrogen bonds between the front and back bases may affect the effect of hydrogen bonds between other bases, while GRU neural network model is just good at dealing with sequence-based problems [18]. Therefore, GRU is chosen to be the main framework of the algorithm in this paper. The algorithm ensures that RNA sequences with different sequence lengths can be accepted by setting the maximum recursion value and a flag marker vector, and solves the problem of imbalance between positive and negative samples by dynamically adjusting the loss function of each base through the *weight* vector.

## Results

**Fig. 1:**
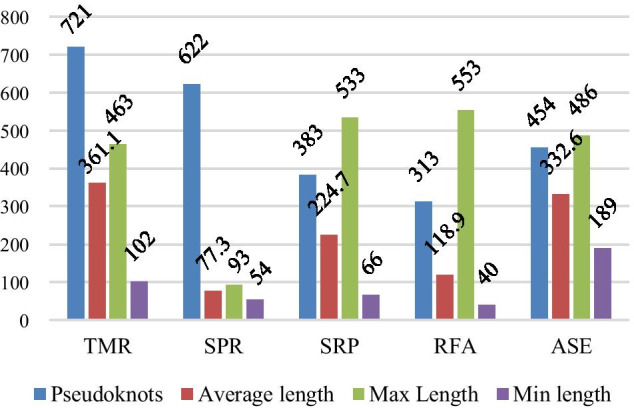
Case of paired and unpaired bases

The prediction results on five data sets based on bidirectional recursive GRU algorithm (GRU in the Table 2.) and variable-length bidirectional recursive GRU algorithm (FLAG in the Table 2.) and VLDB GRU algorithm (VLDB in the Table 2.) are shown in Table 2. From the data in the Table 2, we can see three points of information. First, the three models perform best on SPR dataset and worst on ASE dataset. This result related to the maximum sequence length of the two data sets. As can be seen from the selection of data sets, the maximum sequence length on the SPR data set is 93, and the maximum sequence length on the ASE data set is 486. Since that maximum recursion value obtain by the recursive neural network is the maximum sequence length value of the data set, the greater the maximum sequence length of the data set, the great the maximum recursion value of the recursive neural network and the greater the difficulty in learning the model. Secondly, the variable-length bi-directional recurrent neural network model has better prediction results than the bi-directional recurrent neural network model on five data sets, which shows that the method of introducing a *flag* vector is more reasonable and scientific than the simple method of sequence length supplement, and also makes the learned model more robust and robust. Thirdly, compared with FLAG model, VLDB GRU model is the most prominent in ASE and SRP data sets, and has no obvious advantages in TMR and SPR data sets last time in RFA data sets. Such data results are related to the number of paired bases and unpaired bases on each data set, because VLDB GRU model is an algorithm improvement to solve the problem of imbalance between paired bases and unpaired bases on the data set. As can be seen from Fig. 1, the difference between paired bases and unpaired bases on the five data sets is ASE, SRP, SPR, RFA and TMR from large to small. Among them, the situation of paired bases and unpaired bases on TMR data set is exactly opposite to that of the whole data set. Therefore, the method of introducing a *weight* vector has no obvious advantage on TMR data set, but the VLDB GRU model proposed in this paper can be seen to have a good effect on ASE and SRP data sets. Therefore, based on the above three points, the prediction effect of VLDB model in this paper is better than the other two models.

**Table 2:** Experimental results based on VLDB GRU algorithm

Dataset	Method	SEN	PPV	ACC	MCC
SPR	GRU	0.777	0.687	0.707	0.421
	FLAG	***0.965***	0.853	0.905	0.816
	VLDB	0.962	***0.885***	***0.921***	***0.845***
ASE	GRU	0.983	0.482	0.556	0.323
	FLAG	0.806	0.532	0.645	0.36
	VLDB	***0.826***	***0.652***	***0.727***	***0.475***
RFA	GRU	0.971	0.563	0.661	0.451
	FLAG	0.793	0.613	0.732	0.473
	VLDB	***0.811***	***0.699***	***0.778***	***0.558***
SRP	GRU	0.768	0.676	0.708	0.421
	FLAG	0.798	0.653	0.697	0.408
	VLDB	***0.828***	***0.828***	***0.729***	***0.463***
TMR	GRU	0.974	0.498	0.63	0.434
	FLAG	***0.819***	0.668	***0.769***	***0.543***
	VLDB	0.796	***0.669***	0.765	0.529

The data in bold, italics, underline represent the optimal evaluation index values obtained by different algorithms on the same data set

### Comparison with other algorithms

**Fig. 2:**
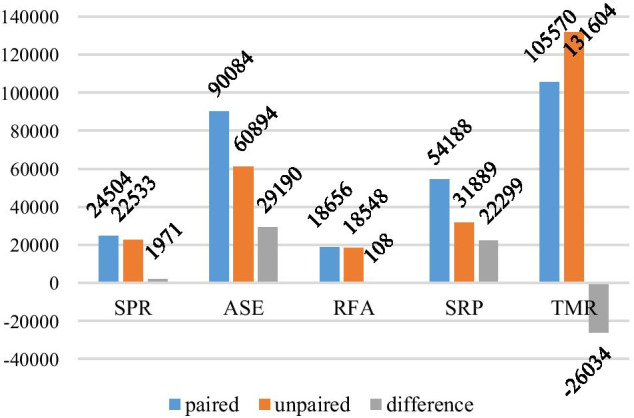
Index difference between VLDB GRU and ProbKnot algorithms

**Fig. 3:**
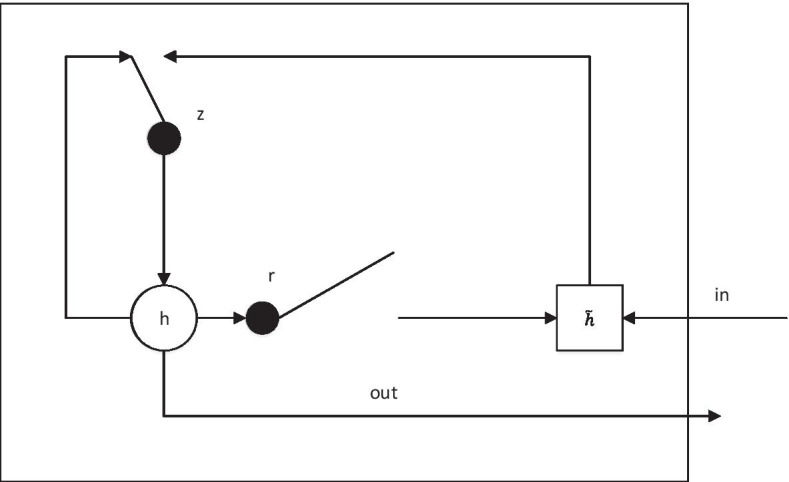
Index Difference between VLDB GRU and SVM algorithms

The training algorithm (VLDB GRU) in this paper is compared with the existing support vector machine algorithm (SVM), ProbKnot algorithm, long shot-term memory (LSTM) algorithm and Cylofold algorithm on 5 data sets SPR, ASE, RFA, SRP and TMR. The experimental comparison results are shown in Table 3. The difference of various indexes in the experiment is shown in Figs. 2 and 3.

**Table 3:** Comparison with other algorithms

Dataset	Method	SEN	PPV	ACC	MCC
SPR	VLDB	***0.962***	***0.885***	***0.921***	***0.845***
	SVM	0.788	0.856	0.834	0.667
	ProbKnot	0.793	0.744	0.772	0.546
	LSTM	0.703	0.71	0.687	0.372
	Cylofold	*	*	*	*
ASE	VLDB	***0.826***	0.652	***0.727***	***0.475***
	SVM	0.712	***0.663***	0.68	0.361
	ProbKnot	0.734	0.564	0.613	0.247
	LSTM	0.81	0.739	0.574	0.786
	Cylofold	0.66	0.575	0.65	0.299
RFA	VLDB	**0.811**	0.699	***0.778***	***0.558***
	SVM	0.151	***0.748***	0.581	0.182
	ProbKnot	0.793	0.555	0.648	0.339
	LSTM	0.794	0.54	0.561	0.141
	Cylofold	0.667	0.551	0.584	0.177
SRP	VLDB	***0.828***	***0.7***	***0.729***	***0.463***
	SVM	0.682	0.566	0.581	0.167
	ProbKnot	0.807	0.598	0.638	0.300
	LSTM	0.824	0.665	0.625	0.123
	Cylofold	0.673	0.563	0.184	0.589
TMR	VLDB	***0.796***	0.669	***0.765***	***0.529***
	SVM	0.498	***0.684***	0.68	0.34
	ProbKnot	0.635	0.388	0.533	0.109
	LSTM	0.85	0.538	0.569	0.18
	Cylofold	0.526	0.433	0.561	0.106

The data in bold, italics, underline represent the optimal evaluation index values obtained by different algorithms on the same data set

*Cylofold algorithm is unable to measure results on SPR data sets with many base deletion sequences

### Case study

**Fig. 4:**
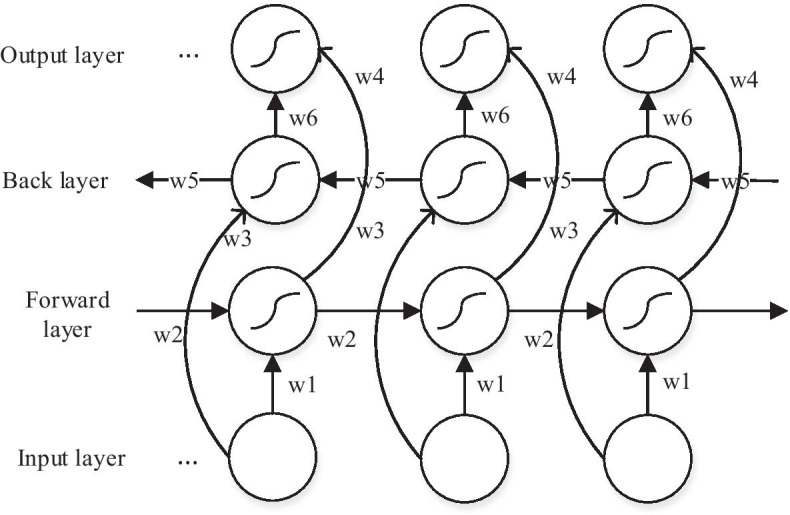
SRP_00256 secondary structure diagrams

SRP_00256(PIR.SPE.) is one of the signal recognition particle ribonucleic acids. Its sequence has a primary length of 93. Such molecules can often recognize more than one codon of the same amino acid. Its 5’ end base is the modified base, A is modified to I(hypopurine), and it can pair with U, C and A. Therefore, the pairing of such RNA is much more complicated. Figure 4 (a) is a natural secondary structure diagram of SRP_00256, (b) is a secondary structure diagram of SRP_00256 predicted herein, and (c) is a secondary structure diagram of SRP_00256 predicted using ProbKnot method. Where black bases indicate correctly predicted paired or unpaired bases, and red indicates incorrectly predicted paired or unpaired bases. Other case study figures can be assessed from http://eie.usts.edu.cn/prj/currentdata/index.html.

## Discussion

As can be seen from Table 3, the VLDB GRU model has obvious advantages over the other two algorithms on the data set SPR. On the one hand, GRU is good at dealing with the problem of correlation between front and back sequences. On the other hand, this model can accept sequences of different lengths without violently truncating the length of the sequences. Because the *weight* vector method also gives the model a better accuracy. In addition, it can be seen that the VLDB GRU model also performs well on the data set ASE, but the index of each algorithm decreases compared with that on the data set SPR, which is largely related to the characteristics of the ASE data set itself, because the proteins on the ASE data set are RNase P proteins, i.e., ribonuclease P, which contain a large number of bases, making the prediction of its secondary structure more difficult. From Figs. 2 and 3, we can see that VLDB GRU model has a great advantage over ProbKnot algorithm, SVM algorithm, LSTM algorithm and Cylofold algorithm in prediction accuracy on each data sets, especially on data set TMR, the proposed method is 13% and 21.9% higher than ProbKnot algorithm in ACC and MCC respectively. Therefore, compared with other algorithms, the VLDB GRU model proposed in this paper can indeed predict the secondary structure of RNA more accurately.

## Conclusion

In this paper, a VLDB GRU model is designed based on the recurrent neural network model. On the one hand, this method improves the traditional processing method for RNA data sets containing sequences of different lengths by setting a *flag* vector for each base in the sequence, i.e. a simple and crude truncation method for the data sets, and effectively uses all information in the protein sequences. On the other hand, the method improves the accuracy of RNA secondary structure prediction. In addition, to solve the problem of imbalance between positive and negative samples, this paper adopts the method of setting a *weight* vector for each base. When calculating the loss function for each base, the proportion of each base in the loss function is dynamically adjusted to avoid the situation that the model falls into local optimization and makes the trained model better. Experiments show that VLDB GRU model improves ACC by 4.7%, 9.1%, 10.4% and 7.7%, respectively, compared with SVM, ProbKnot, LSTM and Cylofold algorithms, which shows that the algorithm proposed in this paper can indeed better predict RNA secondary structure.

## Methods

### Data sets and measurements

**Fig. 5:**
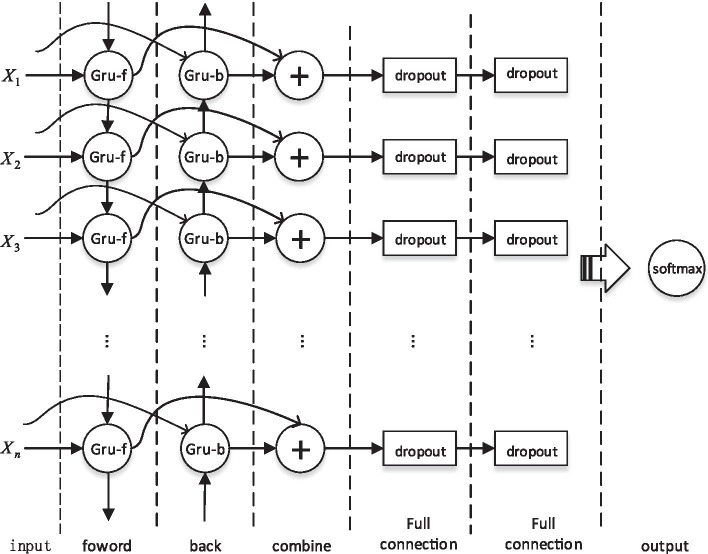
The basic situation of subsets

In this paper, we choose ASE dataset of RNase P type, RFA dataset of Hammerhead Ribozyme type, SPR dataset of Transfer RNA type, TMR dataset of tmRNA type and SPR dataset of Signal Recognition Particle RNA type to predict RNA secondary structure. The reasons are as follows: first, these five RNA data sets are five typical RNA secondary structure prediction data sets; Secondly, these five kinds of RNA data sets all have pseudoknots, which is in line with our research problems; Finally, these five kinds of RNA data sets include the cases that the maximum length of sequences is far greater than the minimum length, the maximum length is close to the minimum length, and the paired bases are larger than, approximately equal to, and smaller than the unpaired bases. These five RNA data sets ensure the persuasiveness of our experimental results [19–21]. The statistics of each subset are shown in Fig. 5, where ‘Pseudoknots’ represent the number of pseudoknots in the dataset, ‘Average length’ represents the average length of the dataset sequence, ‘Max length’ represents the maximum sequence length of the dataset, and ‘Min length’ represents the minimum sequence length of the dataset. The situation of paired bases and unpaired bases in each subset is shown in Fig. 1, where ‘paired’ represents the number of paired bases, ‘unpaired’ represents the number of unpaired bases, and ‘difference’ represents the difference between paired bases and unpaired bases.

In this paper, four indicators are used to evaluate models, i.e., sensitivity (SEN), specificity (PPV), Matthews correlation coefficient (MCC) and accuracy (ACC) to evaluate the model. MCC is an evaluation metrics that combines SEN and PPV [22, 23]. Their calculation methods are shown in Eq. (1).

1 $$\left\{ {\begin{array}{*{20}l} {SEN = \frac{TP}{{TP + FN}} } \hfill \\ {PPV = \frac{TP}{{TP + FP}} } \hfill \\ {MCC = \frac{TP*TN - FP*FN}{{\sqrt {\left( {TP + FP} \right)\left( {TP + FN} \right)\left( {TN + FP} \right)\left( {TN + FN} \right)} }}} \hfill \\ {ACC = \frac{TP + TN}{{TP + TN + FP + FN}}} \hfill \\ \end{array} } \right.$$

where TP represents the number of correctly predicted base pairs; TN means correctly predicting the number of unpaired bases; FP indicates the number of bases predicted to be paired but not actually paired; FN indicates the number of bases predicted to be unpaired but actually paired. The value range of MCC is between -1 and 1, and the value range of the other three indicators is between 0 and 1. The larger these four indicators are, the better the prediction effect of the model is.

The prediction of RNA secondary structure.

Bases are combined units of RNA structure, and stable base pairs are formed by hydrogen bond interaction between bases [24]. Correct prediction of secondary structure is a strong guarantee for prediction of RNA tertiary structure. Pseudoknots are complex and stable structures in many biological cells. Pseudoknots refer to the phenomenon of crossing between paired base pairs, such as base *i* is paired with base *j*, base *m* is paired with base *n*, and the phenomenon that their position sequence number in RNA sequence satisfies *i* < *m* < *j* < *n* is called the existence of pseudoknots in the RNA sequence [25, 26]. Although not all RNA secondary structures have pseudoknots, pseudoknots has an important influence on the function of RNA. Therefore, in order to analyze the real structure of RNA, we must solve the problem of pseudoknots. At present, the prediction of RNA secondary structure with pseudoknots has received extensive attention, which is also a major problem in the prediction of RNA secondary structure.

From the perspective of machine learning, the prediction of RNA secondary structure is to extract the relevant primary information of RNA sequence. After data preprocessing, the structured data is used as the input of machine learning model. Through model training, the matching of each base in RNA sequence can be predicted correctly to the greatest extent. This process can be seen as a supervised learning process with multiple classifications.

### Feature selection and generation

According to Mathews et al. [27], there is a positive correlation between the pairing probability of bases predicted by partition function and the pairing probability of real two bases. Therefore, this paper takes the output result of partition function as a part of the input features to improve the prediction accuracy of RNA secondary structure. In addition, the more frequently a base appears in the sequence, the greater the possibility of pairing with the base. Therefore, in this experiment, the input features are as follows:

1. Through the output of the partition function calculated by RNA structure software, an n*n output matrix will be obtained after a protein sequence with a length of *n* is calculated.
2. The probability that a certain type of base in the sequence appears in the sequence, and the frequency occupied by that type of base in the sequence are recorded and expressed by a one-dimensional vector.
3. Base type information. RNA primary structure can be represented by four bases: A, G, C and U. We use a four-dimensional vector to represent them. Tules are: A-0001, G-0010, C-0100, U-1000, others-0000.

Therefore, for each base, after selecting, transforming and expanding the data features, its input features can be regarded as an (*N* + 5)-dimensions vector, where *N* represents the maximum recursive value of the recurrent neural network, that is, the maximum length of the data set where the sequence is located.

The input of the model is a three-dimensional array *X*[*i*, *j*, *k*], and the first dimension i represents the *i*-th sequence in the data set, with the value range of 1 to batch_size; The second dimension *j* represents the *j*-*th* base of a certain sequence, and its value range is from 1 to *N*. The third dimension *k* represents the *k*-th feature of a base, with a value range of 1 to (*N* + 5). Where batch_size represents the number of training samples of the model each time, the value in this experiment is 200.

The output of the model is a two-dimensional array *Y*, $Y\left[ {i,j} \right] = 0$ means that the (*i* + 1) base in the (*i* + 1)-th sequence is not paired with any base, otherwise it means that the (*j* + 1)-th base and the $y\left[ {i,j} \right]$-th base in the (*i* + 1)-th sequence are paired.

### Bidirectional recursive GRU

GRU is an improved algorithm proposed to overcome the traditional recurrent neural network's inability to handle long-distance dependence well [28, 29]. The algorithm adds two gates, namely update gate and reset gate, whose expressions are as shown in Eq. (2).

2 $$\left\{ {\begin{array}{*{20}l} {r_{t} = \sigma \left( {W_{xr} x_{t} + W_{hr} h_{t - 1} + b_{r} } \right)} \hfill \\ {z_{t} = \sigma \left( {W_{XZ} x_{t} + W_{hz} h_{t - 1} + b_{z} } \right) } \hfill \\ {\widetilde{{h_{t} }} = \tanh \left( {W_{xh} x_{t} + W_{hh} \left( {r_{t} \times h_{t - 1} } \right) + h_{b} } \right)} \hfill \\ {h_{t} = z_{t} \times h_{t - 1} + \left( {1 - z_{t} } \right) \times \widetilde{{h_{t} }} } \hfill \\ \end{array} } \right.$$

**Fig. 6:**
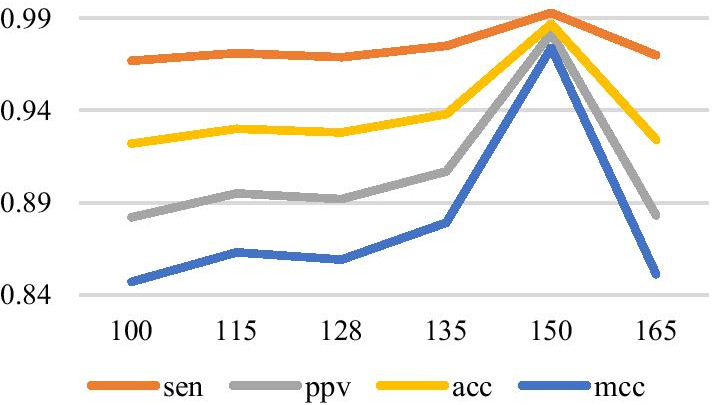
GRU structure

Among them, *x* is the input of the model, and in this paper is the three-dimensional array *X*[*i*, *j*, *k*] described earlier. The input feature of each base corresponds to a recursive cycle, and the total number of recursive cycles is the maximum sequence length *n*. *r* denotes a reset gate, which can decide which information to discard and which new information to add, *z* denotes an update gate, which determines the extent to which previous information is discarded, and $\tilde{h}$ and *h* denote a new hidden state and a current hidden state, respectively. σ and tanh represent Sigmoid function and tanh function, respectively, which are the parameters that the model needs to train and follow. The graphical abstraction is shown in Fig. 6.

Considering that the formation of the secondary structure of RNA is a structure formed by the interaction of hydrogen bonds between bases, the sequence information before and after a base in the RNA sequence will have certain influence on the secondary structure, so the bidirectional recurrent neural network model is selected for training in this experiment. Bidirectional recurrent neural network is a composite recurrent neural network that combines a forward-learning recurrent neural network and a backward-learning recurrent neural network. The calculation process is shown in Eq. (3).

3 $$h_{t} = \overrightarrow {{h_{t} }} + \overleftarrow {{h_{t} }}$$

**Fig. 7:**
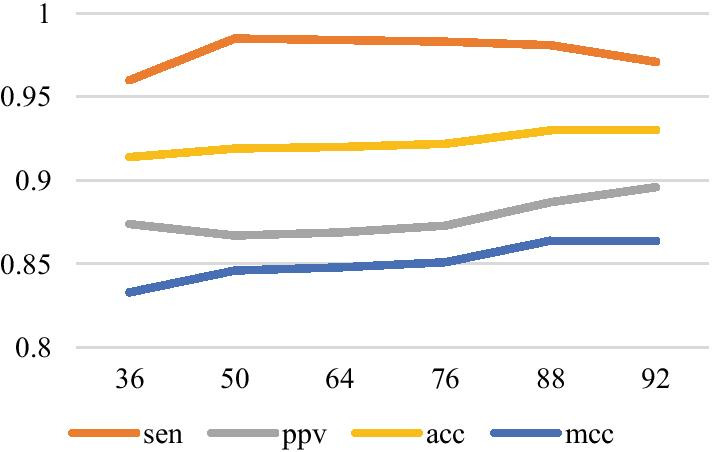
Bidirectional recurrent neural network

The graphical abstraction of the bi-directional recurrent neural network is shown in Fig. 7. The weights *w1* to *w6* in the figure respectively represent the input to the forward and backward hidden layers, the forward and backward hidden layers to the hidden layer itself, and the forward and backward hidden layers to the output layer.

### Variable length bidirectional recursive GRU

Because the length of each RNA sequence is not consistent, the traditional way of truncating the long sequence or simply completing the short sequence will lead to the loss of sequence information, resulting in the waste of information, or adding redundant information to the sequence, both of which have a certain negative impact on the prediction of RNA secondary structure [30–32]. Therefore, in this paper, a flag vector is introduced to carry out zero filling processing on short sequences in the data preprocessing stage, but in the training stage, the filling part will be filtered when the loss function is calculated for each base. Thus not only all effective information of the sequences is utilized, but also the redundant information filled in is not allowed to interfere with the test [33]. The calculation process of the cross entropy of a sequence *m* in the variable-length bidirectional recursive GRU model is as shown in Eqs. (4)–(6).

4 $$cross\_loss_{m} = \frac{{\mathop \sum \nolimits_{i = 1}^{n} flag\left[ i \right] \cdot \,loss_{m} }}{{\mathop \sum \nolimits_{i = 1}^{n} flag\left[ i \right]}}$$

5 $$flag\left[ i \right] = \left\{ {\begin{array}{*{20}l} {1,} \hfill & {i \le n} \hfill \\ {0,} \hfill & {n < i \le N} \hfill \\ \end{array} } \right.$$

6 $$loss_{m} = - \mathop \sum \limits_{j = 1}^{n + 1} y_{{\left[ {i,j} \right]}} \cdot \,\log \left( {y\left[ {i,j} \right]} \right)$$

where the operator “$\cdot$” represents multiplication of the corresponding positions of the vector.

From Eq. (5), it can be found that when *n* < *i* ≤ *N*, *flag*[*i*] takes a value of 0, which makes the values of $flag\left[ i \right] \cdot \,loss_{m}$ also takes a value of 0, i.e. the bases participating in the completion will not affect the value of loss function $cross\_loss_{m}$.

### VLDB GRU

Since the ratio between paired bases and unpaired bases in the RNA STRAND data set is 2:3 [34, 35], in the multi-classification processing mode of this experiment, the pairing number of each base will be given sepecally, so the ratio of the number of bases belonging to each category is 2/n:2/n:…:2/n:3 [36–38]. This is a serious imbalance samples, and the model tends to classify more bases into the category of "unpaired" for higher accuracy [39, 40]. In order to avoid the model falling into this kind of local optimum, this paper sets a weight vector for each base. If the base is a single base and there is no base paired with it, it is assigned 1, otherwise, it is assigned to the sum of all bases in the sequence where the base is located. Thus, the calculation process of the cross entropy of a certain sequence m is shown in Eqs. (7) and (8). The training algorithm of VLDB GRU model is shown in Table 1.

7 $$cross\_loss_{m} = \frac{{\mathop \sum \nolimits_{i = 1}^{n} flag\left[ i \right] \cdot \,weight\left[ i \right] \cdot \,loss_{m} }}{{\mathop \sum \nolimits_{i = 1}^{n} flag\left[ i \right] \cdot \,weight\left[ i \right]}}$$

8 $$weight\left[ i \right] = \left\{ {\begin{array}{*{20}l} {1,} \hfill & {y\_\left[ {i,0} \right] \ne 0} \hfill \\ {\mathop \sum \limits_{i = 1}^{n} y\_[i,0],} \hfill & {y\_[i,0] = 0} \hfill \\ \end{array} } \right.$$

where *y* and *y_* represent the probability that the model predicts a certain category and the label of the sample respectively. The *flag* vector indicates whether a base at the position, 1 indicates existence, 0 indicates inexistence. *n* is the length of the sequence. *y_* is an array of n*(*n* + *1*). When *y_*[*i, j*] is equal to 1, it means that the (*i* + *1*) th base and the (*j*) th base are paired, otherwise it means that they are not paired. When *y_* is equal to 1, it means that the (*i* + 1) th base is not paired with any base.

*x* and *y_* in Table 1 represent the input characteristics of bases and the real labels of bases respectively, and *L*[*i*] corresponds to the loss function of Eq. (7).

### Model parameter setting

**Fig. 8:**
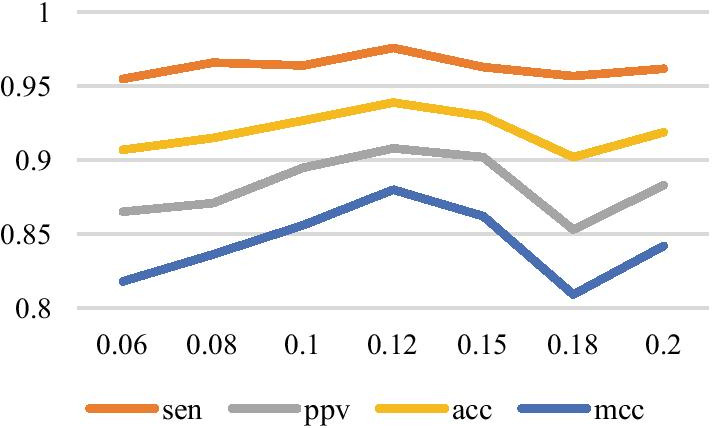
Overall framework of model

The model maps the feature vector corresponding to each base through the input layer to a group of n-dimensional vectors, which are used as the input of the bidirectional GRU model. The output of the bidirectional GRU model is followed by two full connection layers and one output layer to finally classify the data. At the same time, the neural network may fall into over-fitting, so dropout layer is added to solve this problem. The overall design framework of the model is shown in Fig. 8.

**Fig. 9:**
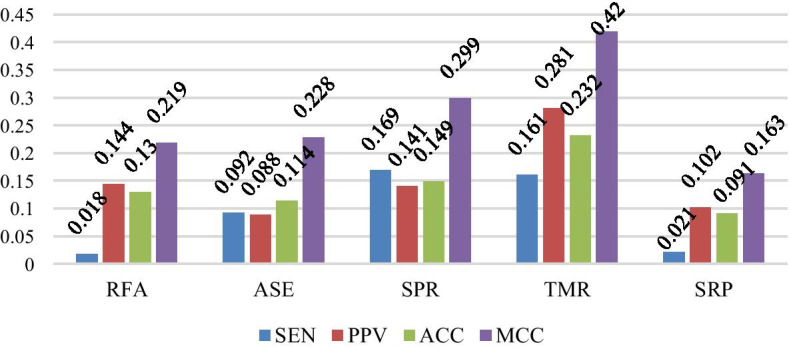
The relationship between each index and the full connection layer

**Fig. 10:**
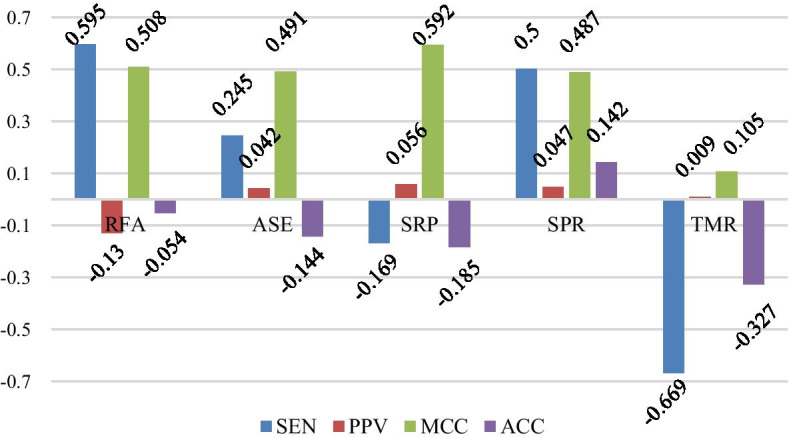
The relationship between each index and hidden layer

**Fig. 11:**
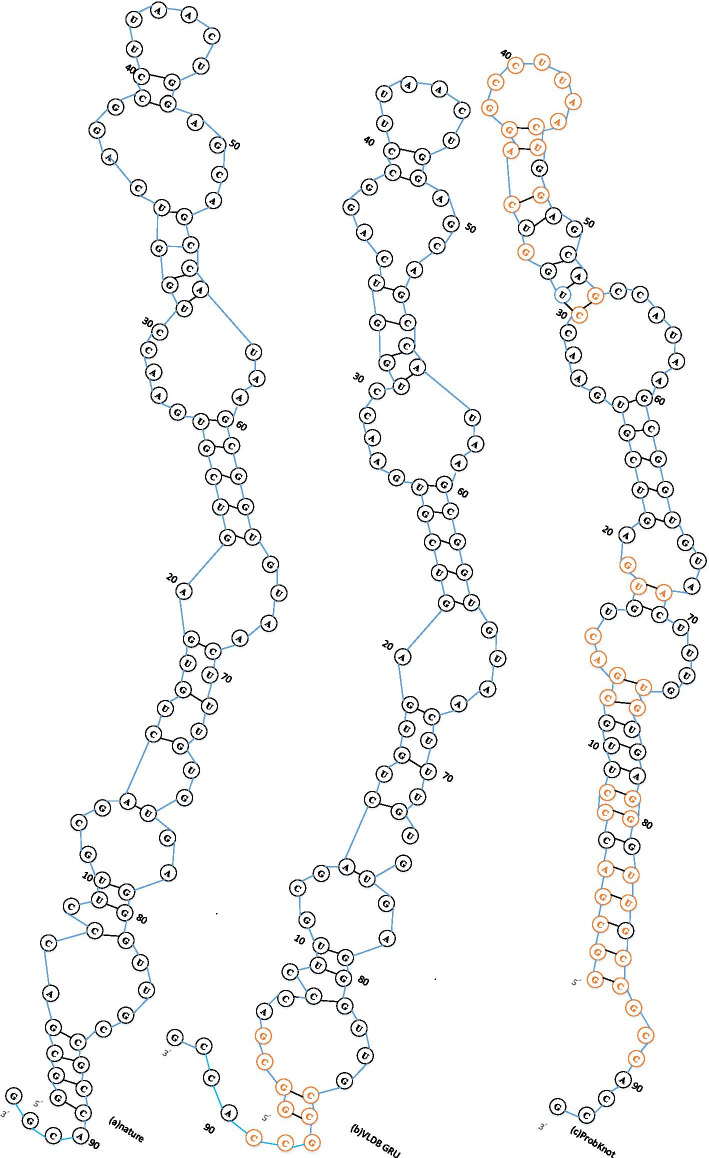
The relationship between various indicators and learning rate

In the VLDB GRU model designed in this paper, variable length means that the lengths of RNA sequences to be predicted are inconsistent. Therefore, we select the maximum sequence length in each data set as the number of GRU recursions. Many experiments show that when the number of GRU hidden layer neurons is selected to be 50, the number of all connected layer neurons is selected to be 150, the learning rate is set to be 0.1, and the maximum number of iterations is set to be 2000, the result is better. The experimental results are shown in Figs. 9, 10, and 11, in which the *y* axis represents the prediction accuracy rate, and the *x* axis represents the set values of the number of layers of the full connection layer, the number of layers of the hidden layer, and the learning rate, respectively.

## Data Availability

The extracted data supporting the conclusions of this article is included within the article. Dataset can be accessed from http://eie.usts.edu.cn/prj/currentdata/index.html.

## Abbreviations

VLDB GRU: Variable-length dynamic bidirectional gated recurrent unit
SVM: Support vector machine
LSTM: Long shot-term memory
